# International Validation of the SULF-FAST Risk-Stratification Tool for Sulfonamide Antibiotic Allergy

**DOI:** 10.1001/jamanetworkopen.2025.19113

**Published:** 2025-07-07

**Authors:** Florian Stehlin, Sara Vogrin, Elise Mitri, Ghislaine A. C. Isabwe, Jason A. Trubiano, Ana-Maria Copaescu

**Affiliations:** 1 Division of Allergy and Clinical Immunology, Department of Medicine, McGill University Health Center (MUHC), McGill University, Montreal, Quebec, Canada; 2 Division of Immunology and Allergy, Department of Medicine, Lausanne University Hospital, Lausanne, Switzerland; 3 The Research Institute of the McGill University Health Centre, McGill University, McGill University Health Centre (MUHC), Montreal, Quebec, Canada; 4 Center for Antibiotic Allergy and Research, Department of Infectious Diseases, Austin Health, Heidelberg, Victoria, Australia; 5 Department of Infectious Diseases, at the Peter Doherty Institute for Infection and Immunity, Melbourne Medical School, University of Melbourne, Victoria, Australia

## Abstract

This cohort study evaluates the validity of the SULF-FAST risk-stratification tool for sulfonamide antibiotic allergy and describes outcomes in patients with direct oral challenges.

## Introduction

Safe re-exposure in patients allergic to sulfonamide antibiotic (SA) previously relied on resource-intensive procedures such as desensitization.^1^ Direct oral challenge (DOC) has recently been used to delabel low-risk patients.^2,3^ The SULF-FAST clinical decision rule has been proposed for risk stratification.^4^ Our study aimed to externally validate this tool and describe DOC outcomes in inpatients and outpatients.

## Methods

In this multicenter cohort study, adults with SA allergy were prospectively recruited at the McGill University Health Centre (MUHC, Canada) and at Austin Health (AH, Australia) from April 2022 to May 2024. Patients were evaluated upon request at MUHC’s drug allergy clinic or proactively as inpatients at AH. The AH and MUHC ethics committees approved the study, and informed written consent was obtained. This manuscript followed Strengthening the Reporting of Observational Studies in Epidemiology (STROBE) reporting guidelines for cohort studies. Following risk stratification with the SULF-FAST tool, patients with a score of 0 to 2 had DOC to cotrimoxazole (Co-T) (80 mg trimethoprim/400 mg sulfamethoxazole at AH; 160 mg trimethoprim/800 mg sulfamethoxazole at MUHC). Co-T skin testing could be performed before the challenge if the SULF-FAST score was 3 to 5 (eMethods in Supplement 1). Severe cutaneous adverse reactions were a contraindication to challenge. An allergy was confirmed following a positive allergy workup (ie, a positive Co-T skin test or oral challenge).

Analysis was performed using Stata version 18 (StataCorp LLC), including Fisher exact test for group comparisons and calculations of diagnostic performance. A 2-sided *P *value less than .05 was deemed significant.

## Results

Among the 125 patients assessed (96 at MUHC and 29 at AH), the median (IQR) age was 60 (45-67) years, 91 of 125 (72.8%) were female, and 36 of 125 (38.8%) were immunocompromised. Thirty-one (24.8%) were inpatients. Regarding the index reaction, a diffuse rash was reported by 64 of 125 patients (51.2%), anaphylaxis by 5 of 125 (4.0%), and angioedema by 8 of 125 (6.4%). The reaction occurred in childhood for 32 of 125 patients (25.6%) and was unknown for 17 of 125 (13.6%) (Table). Most patients had a SULF-FAST score of 0 to 1 (98 of 125 patients [78.4%]) (Figure).

**Table.** Population Characteristics

Characteristic	Patients, No. (%)		
	Total (N = 125)	MUHC (n = 96)	AH (n = 29)
Age at assessment, y, median (IQR)	60 (45-67)	58 (44-66)	64 (56-69)
Sex			
Female	91 (72.8)	76 (79.0)	15 (52.0)
Male	34 (27.2)	20 (21.0)	14 (48.0)
Inpatient	31 (24.8)	2 (2.1)	29 (100.0)
Immunocompromised patient	36 (38.8)	22 (22.9)	14 (48.3)
Index reaction^a^			
Diffuse rash	64 (51.2)	54 (56.0)	10 (34.0)
Urticaria	17 (13.6)	13 (14.0)	4 (14.0)
Respiratory distress	5 (4.0)	5 (5.0)	0
Angioedema	8 (6.4)	8 (8.0)	0
Laryngeal involvement	5 (4.0)	5 (5.0)	0
Anaphylaxis	5 (4.0)	5 (5.0)	0
Hepatic impairment	1 (0.8)	1 (1.0)	0
Unknown reaction	17 (13.6)	13 (14.0)	4 (14.0)
Childhood (<12 y)	32 (25.6)	21 (22.0)	11 (38.0)
Allergy assessment			
Skin test performed	18 (14.4)	18 (19.0)	0
Positive skin test	6 (4.8)	6 (6.0)	0
Oral challenge performed	119 (95.2)	90 (94.0)	29 (100.0)
Positive challenge	7 (5.6)	7 (7.0)	0
SULF-FAST components			
Treatment required for reaction	91 (72.8)	73 (76.0)	18 (62.0)
Anaphylaxis, angioedema, or SCAR	12 (9.6)	12 (12.0)	0
≤5 y since reaction	15 (12.0)	15 (16.0)	0

Abbreviation: SCAR, severe cutaneous adverse reactions.

^a^ These categories are not mutually exclusive.

**Figure.**
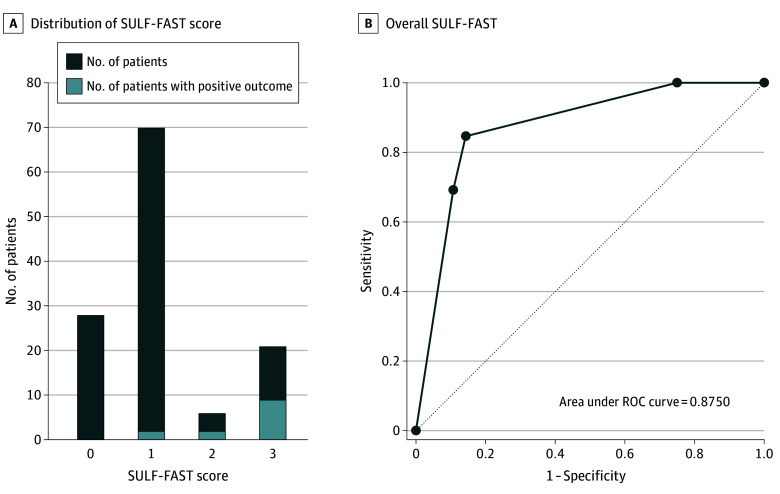
SULF-FAST Performance ROC indicates receiver operating characteristic.

An allergy was excluded in 112 of 125 patients (89.6%; 95% CI, 82.9%-94.3%). DOC was negative in 100 of 104 patients (96.1%) with a score less than 3, all others (4 of 104 patients [3.9%]; ; among them 2 of the 6 patients with a score of 2 [33.3%]) had a mild reaction (3 had a skin rash that resolved within 24 hours with antihistamines; among them, 1 also had joint pain responding to ibuprofen, and the last patient had a 2-day fever responding to acetaminophen). In comparison, 9 of 21 patients (42.9%) with a score of 3 had positive testing (6 via positive skin test and 3 via positive OC). A reaction more than 5 years before or during childhood (≤12 years) was significantly associated with negative testing (69.2% vs 5.4%; *P* < .01 and 0.0% vs 28.6%; *P* = .04, respectively). The Figure summarizes the performance of the SULF-FAST tool, with a clear association between the score and the rate of reaction and with high negative predictive value and area under the curve.

## Discussion

We prospectively validated the SULF-FAST tool in an international multicenter cohort, including inpatients and outpatients with a high area under the curve and negative predictive value. A score less than 3 stratified patients to safely receive a Co-T DOC, which was negative in over 95% with no serious adverse events. The time since the reaction was a key element in determining the risk of a positive test. The performance of SULF-FAST was similar to that of PEN-FAST, a tool validated for penicillin allergy.^5^

A limitation of our study remains the small number of patients and the high prevalence of low-risk patients. Only patients with a score of 3 or more had skin testing, inducing a possible bias toward positive testing. Also, the number of patients with a score of 2 (strictly) was limited, and the proportion of positive testing was significant (33.3%). In addition, patients with type III, serum-sickness-like reactions (2 of 2 patients) reacted in our cohort, and we recommend not assessing these with SULF-FAST. Lastly, in the context of no validated challenge protocol for Co-T delayed reactions, we performed 1-dose challenges.

To our knowledge, this is the largest prospective external validation of an SA allergy risk-stratification tool. This study provides evidence for implementing SULF-FAST to enable SA use.^6^ However, its applicability by nontrained medical personnel needs to be further evaluated.

## Notes

Supplement 1. **eMethods.** Management Protocol

Supplement 2.
Data Sharing Statement
