# Supplementary material for: International Validation of the SULF-FAST Risk-Stratification Tool for Sulfonamide Antibiotic Allergy
Source: JAMA Netw Open. 2025 Jul 7;8(7):e2519113. doi: 10.1001/jamanetworkopen.2025.19113 (PMC12235491; doi:10.1001/jamanetworkopen.2025.19113)
Supplement: Supplement 1. — eMethods. Management Protocol [file jamanetwopen-e2519113-s001.pdf]

Supplemental Online Content

Stehlin F, Vogrin S, Mitri E, Isabwe GAC, Trubiano JA, Copaescu A-M. International validation of the SULF-FAST risk-stratification tool for sulfonamide antibiotic allergy. *JAMA Netw Open*. 2025;8(7):e2519113. doi:10.1001/jamanetworkopen.2025.19113

eMethods. Management Protocol

This supplemental material has been provided by the authors to give readers additional information about their work.

© 2025 Stehlin F et al. *JAMA Network Open*.

eMethods. Management protocol

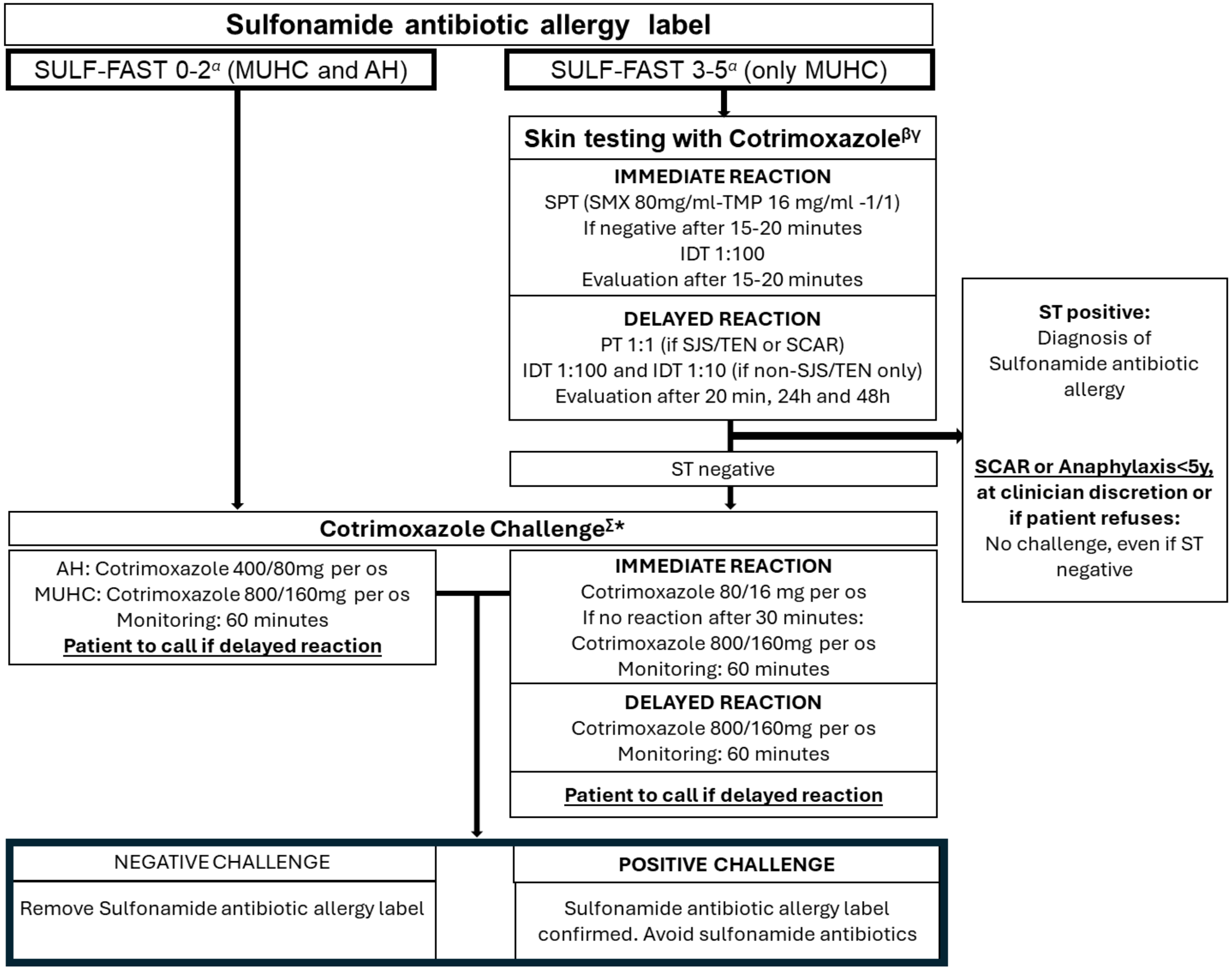

Legend:
[α]First step of the management: risk-stratification with SULF-FAST based on index reaction
[β]At clinician discretion
[γ]Skin Tests on 3 volunteers:
- *SPT and IDT 1/100: Non irritant*
- *IDT 1/10:*
  - *Irritant in all 3*
  - *No delayed reactions*

[Σ] Challenge performed at clinician discretion and after explaining benefits and risks to the patient. SCAR is a contra-indication to challenge.
**Placebo given 30 minutes before all challenges at MUHC*

Abbreviations: MUHC: McGill University Health Centre, AH: Austin Health, SMX: Sulfamethoxazole, TMP: Trimethoprim, SPT: skin prick test, IDT: intradermal test, PT: patch test, SJS/TEN: Stevens-Johnson syndrome/toxic epidermal necrolysis , SCAR: severe cutaneous adverse reaction, y: years

© 2025 Stehlin F et al. *JAMA Network Open*.
