# Supplementary material for: International Validation of the SULF-FAST Risk-Stratification Tool for Sulfonamide Antibiotic Allergy
Source: JAMA Netw Open. 2025 Jul 7;8(7):e2519113. doi: 10.1001/jamanetworkopen.2025.19113 (PMC12235491; doi:10.1001/jamanetworkopen.2025.19113)
Supplement: Supplement 2. — Data Sharing Statement [file jamanetwopen-e2519113-s002.pdf]

Data Sharing Statement

Stehlin. International Validation of the SULF-FAST Risk-Stratification Tool for Sulfonamide Antibiotic Allergy. *JAMA Netw Open*. Published July 07, 2025.
doi:10.1001/jamanetworkopen.2025.19113

Data
Data available: No

Additional Information
Explanation for why data not available: Coded data may be available upon reasonable request to the corresponding author
